# HTLV-1 Evades Type I Interferon Antiviral Signaling by Inducing the Suppressor of Cytokine Signaling 1 (SOCS1)

**DOI:** 10.1371/journal.ppat.1001177

**Published:** 2010-11-04

**Authors:** Stéphanie Olière, Eduardo Hernandez, Agnès Lézin, Meztli Arguello, Renée Douville, Thi Lien-Anh Nguyen, Stéphane Olindo, Gérard Panelatti, Mirdad Kazanji, Peter Wilkinson, Rafick-Pierre Sékaly, Raymond Césaire, John Hiscott

**Affiliations:** 1 Molecular Oncology Group, Lady Davis Institute for Medical Research, Jewish General Hospital, Montreal, Quebec, Canada; 2 Departments of Microbiology and Medicine, McGill University, Montreal, Quebec, Canada; 3 Laboratoire de Virologie-Immunologie, Service de Neurologie and JE2503, Université des Antilles et de la Guyane, Centre Hospitalier Universitaire de Fort-de-France, Fort-de-France, Martinique; 4 Unité de Rétrovirologie, Centre International de Recherches Médicales de Franceville (CIRMF), Franceville, Gabon; 5 Laboratoire d'Immunologie, Centre de Recherche du Centre Hospitalier de l'Université de Montréal Saint-Luc, Montréal, Quebec, Canada; University of Pennsylvania School of Medicine, United States of America

## Abstract

Human T cell leukemia virus type 1 (HTLV-1) is the etiologic agent of Adult T cell Leukemia (ATL) and the neurological disorder HTLV-1-associated myelopathy/tropical spastic paraparesis (HAM/TSP). Although the majority of HTLV-1–infected individuals remain asymptomatic carriers (AC) during their lifetime, 2–5% will develop either ATL or HAM/TSP, but never both. To better understand the gene expression changes in HTLV-1-associated diseases, we examined the mRNA profiles of CD4+ T cells isolated from 7 ATL, 12 HAM/TSP, 11 AC and 8 non-infected controls. Using genomic approaches followed by bioinformatic analysis, we identified gene expression pattern characteristic of HTLV-1 infected individuals and particular disease states. Of particular interest, the suppressor of cytokine signaling 1—SOCS1—was upregulated in HAM/TSP and AC patients but not in ATL. Moreover, SOCS1 was positively correlated with the expression of HTLV-1 mRNA in HAM/TSP patient samples. In primary PBMCs transfected with a HTLV-1 proviral clone and in HTLV-1-transformed MT-2 cells, HTLV-1 replication correlated with induction of SOCS1 and inhibition of IFN-α/β and IFN-stimulated gene expression. Targeting SOCS1 with siRNA restored type I IFN production and reduced HTLV-1 replication in MT-2 cells. Conversely, exogenous expression of SOCS1 resulted in enhanced HTLV-1 mRNA synthesis. In addition to inhibiting signaling downstream of the IFN receptor, SOCS1 inhibited IFN-β production by targeting IRF3 for ubiquitination and proteasomal degradation. These observations identify a novel SOCS1 driven mechanism of evasion of the type I IFN antiviral response against HTLV-1.

## Author Summary

Infection with HTLV-1 leads to the development of Adult T cell Leukemia (ATL) or the neurological disorder HTLV-1-associated myelopathy/tropical spastic paraparesis (HAM/TSP). Although the majority of HTLV-1–infected individuals remain asymptomatic carriers (AC) during their lifetime, 2–5% will develop either ATL or HAM/TSP. Using gene expression profiling of CD4+ T lymphocytes from HTLV-1 infected patients, we identified Suppressor of cytokine signaling 1 (SOCS1) as being highly expressed in HAM/TSP and AC patients. SOCS1 expression positively correlated with the high HTLV-1 mRNA load that is characteristic of HAM/TSP patients. SOCS1 inhibited cellular antiviral signaling during HTLV-1 infection by degrading IRF3, an essential transcription factor in the interferon pathway. Our study reveals a novel evasion mechanism utilized by HTLV-1 that leads to increased retroviral replication, without triggering the innate immune response.

## Introduction

Infection with the Human T cell Leukemia Virus type I (HTLV-I) can result in a number of disorders, including the aggressive T cell malignancy Adult T cell Leukemia (ATL) and the chronic, progressive neurologic disorder termed HTLV-1-associated myelopathy/tropical spastic paraparesis (HAM/TSP) [1], [2], [3]. In endemic areas including Southern Japan, the Caribbean basin, Western Africa and Central/South America - where infection rates range from 2 to 30%- these diseases are major causes of mortality and morbidity [4]. The majority of HTLV-1–infected individuals remain asymptomatic (AC) during their lifetime and only ∼2–5% of AC will develop either ATL or HAM/TSP [5], [6]. Although the factors determining progression from AC to ATL or HAM/TSP remain unknown, it is well established that the risk of ATL vs. HAM/TSP development varies dramatically with the geographical distribution of HTLV-1-infected populations.

Clinically, acute ATL is characterized by abnormally elevated T cell counts, accompanied by readily observed ‘flower cells’ – multi-lobed, leukemic cells with highly condensed chromatin - hypercalcemia, prominent skin lesions, hepatosplenomegaly and suffer from serious bacterial, viral, fungal and protozoan infections. Most patients present at this final acute stage, often unaware of their HTLV-1 positive status and given a poor prognosis, with a survival estimate of 6–10 months [7]. Transformation of CD4+ T lymphocytes by HTLV-1 and the development of ATL leukemogenesis generally occur in two stages [8], [9]. After infection with the blood borne pathogen, HTLV-1 induces IL-2-dependent, CD4+ T cell proliferation, that over a period of decades *in vivo*, progresses with the emergence of an IL-2-independent malignant clone that has accumulated multiple secondary genetic changes in growth regulatory and tumor suppressor genes [9], [10]. HTLV-1 encodes the 40-kDa nuclear oncoprotein Tax that promotes cellular transformation through dysregulation of mitotic checkpoints, activation of cellular signaling pathways and inactivation of tumor suppressors (reviewed in [11], [12]).

HAM/TSP is a systemic immune-mediated inflammatory disease characterized by demyelination of motor neurons in the spinal cord, although other tissues can also be damaged [13]. HAM/TSP attacks in the prime of life (median age of onset: 35 years) and is associated with a clinical history that includes neurological symptoms in 80% of cases – gradual onset of leg weakness, paresthesis, and impairment of urinary or bowel function. Central nervous system (CNS) white matter lesions of the spinal cord harbor activated CD4^+^ and CD8^+^ T cells during early stages of disease, with a predominance of CD8^+^ T cells later in disease. HTLV-1 viral RNA has been found associated with CD4^+^ T cells and astrocytes in CNS lesions, suggesting that virus-infected cells migrate through the blood-brain barrier and infect CNS resident cells [14], [15]. While the mechanisms resulting in HAM/TSP development remain unresolved, it has been suggested that Tax expression in CNS cells triggers a strong virus-specific CD8^+^ (as well as CD4^+^) T cell response leading to inflammation, myelin loss, and axonal damage [16], [17]. Elevated levels of proinflammatory cytokines (IL-6, IFN-γ, IL-15, IL-1β, TNF-α and IL-12) have been detected in the serum and cerebrospinal fluid (CSF) of patients with HAM/TSP, corroborating the link between HAM/STP development and dysregulated inflammation [18], [19].

It is widely accepted that type I interferon (IFN-α/β) has a negative impact on HIV-1 replication [20], [21], and although few reports have documented the IFN antiviral effects during HTLV-1 infection, type I IFN constitutes a potent anti-retroviral mechanism that affects HTLV-1 replication [22], [23]. In return, HTLV-1 infection of pDCs results in impaired IFN-α production, and correlates with elevated HTLV-1 proviral load in infected individuals [24]. Central to the establishment of an antiviral state is the activation of diverse IFN-stimulated genes (ISGs) which restrict viral replication [25]. Interferon regulatory factors IRF3 and IRF7 play essential roles in the early phase of IFN gene activation [26]. IRF3 is constitutively expressed and is activated by C-terminal phosphorylation by IKKε and TBK1, which promotes transactivation of downstream genes such as IFN-β and IFN-α [27], [28]. In contrast, IRF7 protein is synthesized *de novo* upon IFN stimulation and contributes to the amplification of the IFN response, *via* expression of multiple IFN-α subtypes [29]. IRF-driven IFN secretion acts in a paracrine fashion to induce the expression of hundreds of genes through engagement of the IFN receptors and activation of the JAK/STAT signaling pathway, which leads to the development of an antiviral state (reviewed in [30], [31]).

IFN-induced JAK/STAT signaling is negatively regulated at different levels by several cellular factors to control the extent of the antiviral response and limit tissue damage [32], [33]. Suppressor of cytokine signaling 1 (SOCS1) belongs to the SOCS protein family and is induced after virus infection [34]. SOCS1 suppresses IFN signaling by direct binding to phosphorylated type I IFN receptor and active JAK kinase, abrogating phosphorylation of STAT1 [35]. Through its SOCS-Box domain, SOCS1 targets various proteins such as JAK, MAL, p65, Steel, Vav for proteasomal degradation [36], [37], [38]. The SOCS-Box serves as a recruiting platform for the formation of a E3 ligase complex composed of elongin B/C-Cullin 2 and Rbx2 [39], [40]. Thus, SOCS1 initiates and orchestrates the events leading to proteasomal degradation of target proteins [34]. Recently, virus-induced upregulation of SOCS1 protein has emerged as a novel mechanism employed by several viruses to evade the antiviral response [41], [42], [43]. In the present study, global gene expression profiles in CD4+ T lymphocytes were examined in a unique cohort of 30 HTLV-1 infected individuals from the Caribbean basin including ATL, HAM/TSP and asymptomatic carriers (AC) patients. Interestingly, among the many genes dysregulated in HTLV-1 infected patients, SOCS1 was highly expressed in CD4+ T cells from HAM/TSP and AC patients, but not in ATL. Subsequent biochemical analysis demonstrated that HTLV-1-induced SOCS1 expression played a positive role in viral replication through inhibition of the IFN response. SOCS1 directly interacted with IRF3 and promoted its proteasomal degradation in a SOCS-Box dependent manner, thus identifying a novel mechanism of HTLV-1 mediated evasion of the IFN response.

## Results

### Gene expression profiling of CD4+ T cells from ATL, HAM/TSP and AC patients

**Figure 1:**
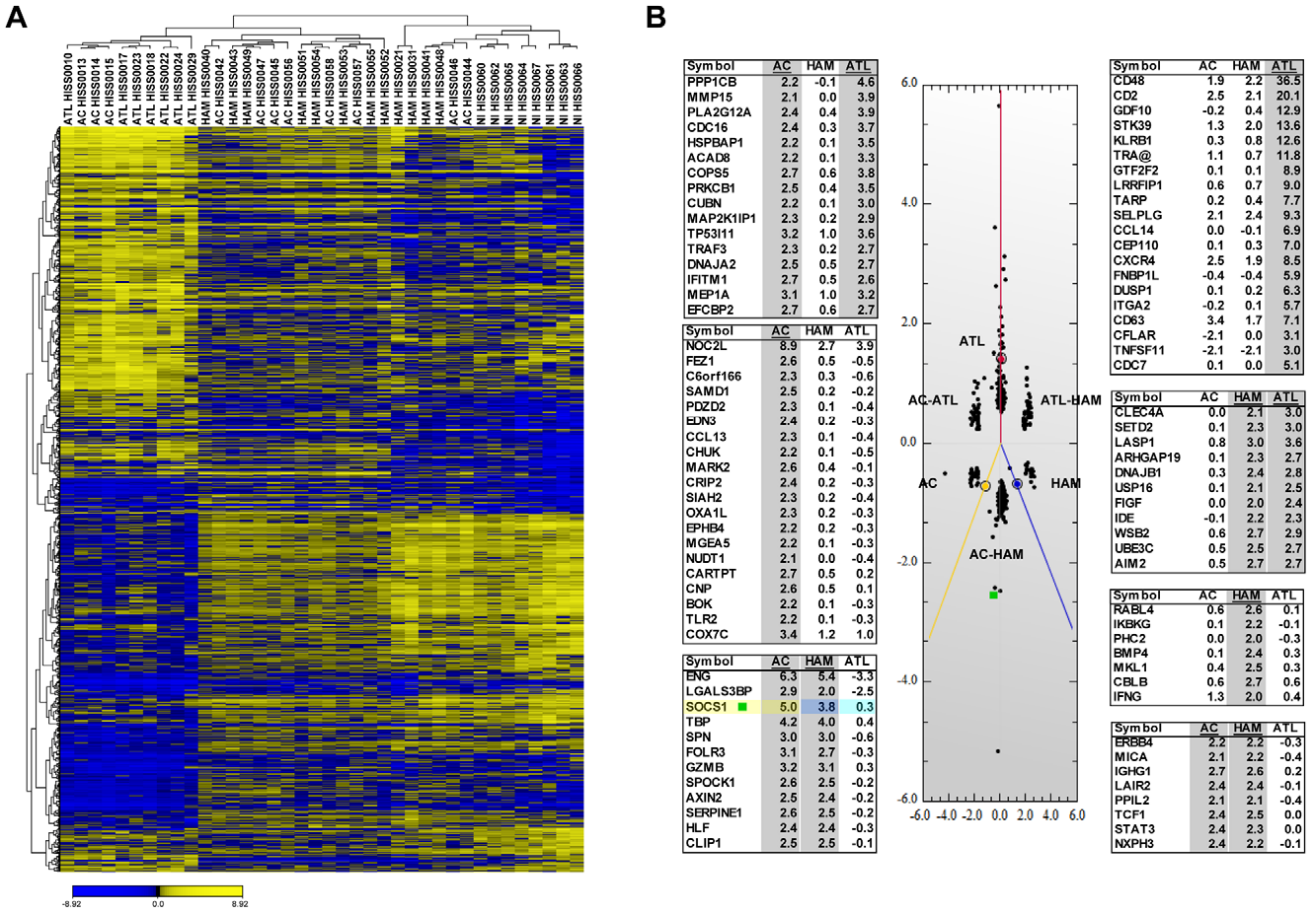
Expression profiling of genes differentially expressed in HTLV-1 associated-diseases. (A) Unsupervised hierarchical clustering of the 1039 genes differentially expressed in CD4+ T lymphocytes from 7 ATL, 12 HAM/TSP, 11 AC and 8 NI donors. Significant variation in the expression pattern of 1039 genes was determined by ANOVA (*p*<0.01). Each row represents the relative level of expression for a single gene; each column shows the expression level for a single sample. The yellow and blue colors indicate high and low expression, respectively. Genes were clustered into 3 groups using a complete linkage and Pearson correlation as distance metric. (B) Pair-wise contrast by Correspondance Analysis (PCA)**.** PCA analysis was performed on the 1039 genes selected by ANOVA. Correspondence analysis shows genes plotted in discriminate space and separated into 6 groups: genes associated specifically with ATL, HAM/TSP or AC, and genes commonly regulated in the three groups; AC-HAM/TSP, AC-ATL or ATL-HAM/TSP. Colored lines denote the direction of class medians and black dots correspond to the genes. Genes with high differential expression in a group are located far-off the center (gene expression of NI), in the direction determined by the group. The closest to the group line, the more evident is the associations of the genes with that group. Genes that are down-regulated in this group appear on the opposite site of the centroid. A short list of the top genes was created by selecting the genes having ≥2 fold changes relative to NI. The genes lists are ranked by the absolute variation (Max-Min) in fold change expression among the three HTLV-1 infected groups.

To analyze gene expression profiles of CD4+ T cells isolated from HTLV-1 infected patients, we gathered a unique cohort of 30 HTLV-1 infected individuals from the Caribbean basin, including 11 AC, 7 ATL, 12 HAM/TSP and 8 healthy, non-infected donors (NI) (Table S1). Microarray experiments were performed using the human ImmuneArray cDNA array (UHN Microarray Center, University of Toronto), followed by higher order analysis. About three thousand genes were analyzed with Future Selection Subset/ANOVA on log-transformed data, followed by unsupervised hierarchical clustering on 1039 genes selected by Anova analysis (*p*<0.01) (Figure 1A). These genes displayed differential expression patterns depending on the type of HTLV-1-associated disease. Unsupervised clustering based on the 1039 genes signature accurately discriminated between NI, HAM/TSP and ATL patients. AC samples however did not separate as an individual cluster, but rather distributed amongst HAM/TSP and ATL samples. Also, two of the HAM/TSP patients and two AC clustered with the NI group, suggesting that the profile of their circulating CD4+ T lymphocytes had not undergone significant variation compared to healthy donors.

Pair-wise correspondance analysis (PCA) was performed on the top 500 genes modulated in HTLV-1-infected versus non-infected samples (*p* value <0.01, false discovery rate (FDR)  = 0.17%) (Figure 1B). PCA identified prevalent expression profiles among the three clinical groups, and confirmed significant class discrimination between non-HTLV-1-infected donors (NI) versus each of the HTLV-1-associated diseases when plotted in two dimensions (Figure 1B). Gene clusters common to each HTLV-1-infected clinical group, and shared within pair-wise comparisons (AC-HAM/TSP, AC-ATL and ATL-HAM/TSP), could be identified and are presented in the adjoining Tables of Figure 1B. For each grouping, genes with a high differential expression are located with quantitative spacing from the center comparator (gene expression of NI group). Specifically, SOCS1 (green square) was identified as a strongly upregulated gene in both HAM/TSP and AC patients, in agreement with the prior observation by Nishiura *et al.* [44]. Since SOCS1 is known to counter-regulate the anti-viral response, it was selected as a gene of interest for further study.

### HTLV-1 infection induces the expression of SOCS1 mRNA

**Figure 2:**
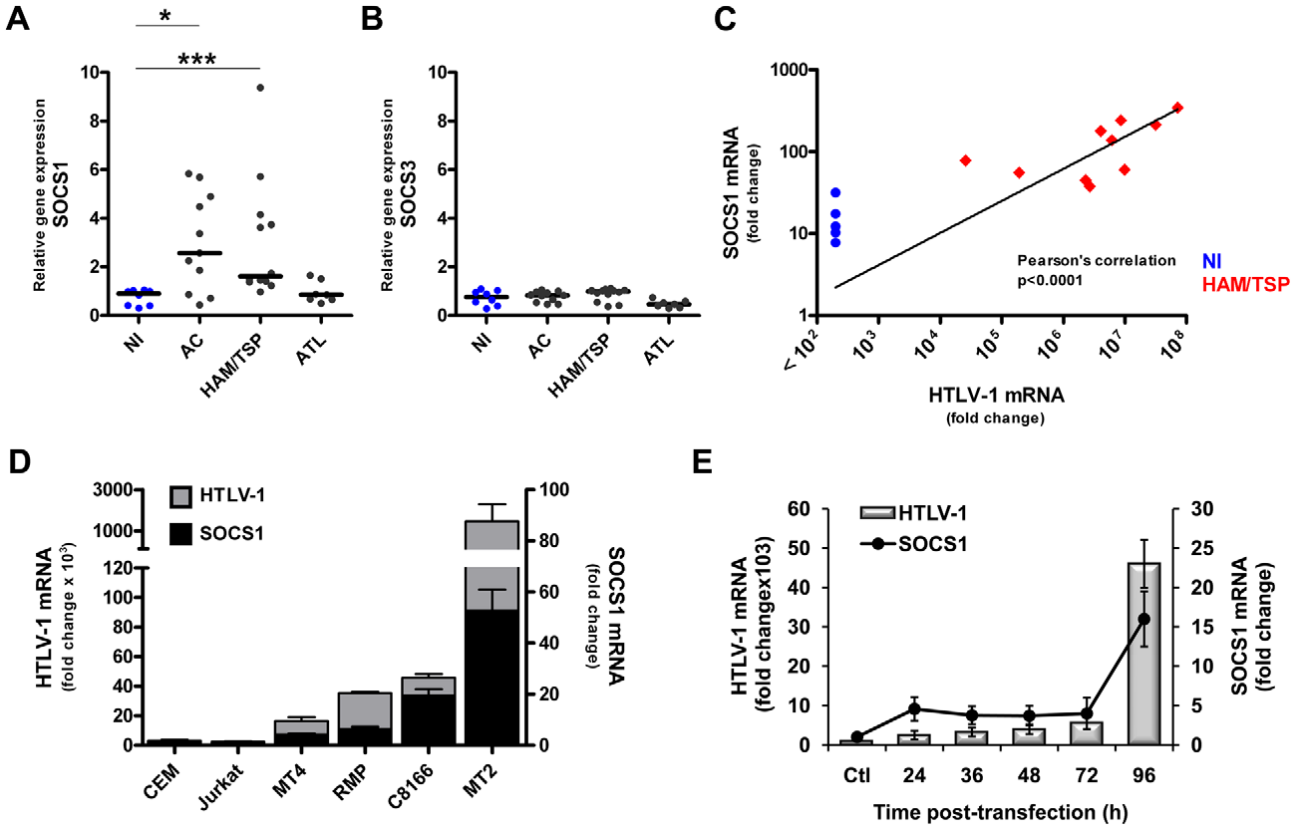
HTLV-1 infection results in the induction of SOCS1 mRNA levels in *ex vivo* CD4+ T cells and correlates with HTLV-1 mRNA expression. (A, B) Comparison of mRNA expression of SOCS1 and SOCS3 in CD4+ T cells from HTLV-1 infected and NI patients. CD4+ T cells were isolated from PBMCs of HTLV-1 infected and NI patients; SOCS1 (A) and SOCS3 (B) mRNA levels were assessed using a human cDNA array. *SOCS1* gene expression was stratified by clinical status forming 4 groups: NI, ATL, AC and HAM/TSP. Each point represents SOCS level from one individual, with black bars showing median value in each group. Mann-Whitney test was used to compare intensity of SOCS expression between groups (*, *p*<0.05; ***, *p*<0.001). (C) Expression of SOCS1 mRNA levels correlated with HTLV-1 mRNA load in HAM/TSP patients. CD4+ T cells from HAM/TSP and NI patients were lysed and total RNA was subjected to reverse transcription. cDNA was analyzed by quantitative real time PCR to assess mRNA levels of SOCS1 and HTLV-1 (Pearson's correlation p<0.0001, line represents log-log non-linear fit of the data) (D) Expression of SOCS1 and HTLV-1 mRNAs was measured in HTLV-1-carrying T cell lines (MT-2, MT-4, C8166, RMP) vs. HTLV-1-negative T cell lines (CEM, Jurkat). Cells were treated as previously and cDNA was analyzed by quantitative real time PCR to measured *SOCS1* and HTLV-1 gene expression. Equivalent mRNA amounts were normalized to *GAPDH* gene expression and calculated as fold change with the levels of uninfected CEM cells set arbitrarily as 1. (E) PBMCs from healthy individuals were electroporated with either HTLV-1 provirus (pX1M-TM) or control empty vector (ctl). At the indicated times, total RNA was extracted and analyzed for *SOCS1* and HTLV-1 gene expression. Equivalent mRNA amounts were normalized to GAPDH mRNA expression and calculated as fold change from the levels of control cells that were arbitrarily set as 1.

Efficient HTLV-1 spread must overcome cellular antiviral programs [45]; yet how HTLV-1 evades the host innate immune response is poorly understood. SOCS1 stood out among the many genes identified as having the potential to counteract the innate immune response against HTLV-1. HAM/TSP and AC patients exhibited a greater than two fold increase in *SOCS1* gene expression compared to NI individuals (Fisher's test, *p* value  = 0.054 and <0.05, respectively) (Figure 2A). However, no significant difference in mRNA levels of SOCS1 was found between ATL and NI patients, suggesting that SOCS1 expression was upregulated in AC and HAM/TSP. This increase was specific for SOCS1, as SOCS3 mRNA was unchanged in HTLV-1 infected samples compared to control samples (fold change <2) (Figure 2B). Using a separate cohort of patient samples (Table S2), we demonstrated that SOCS1 expression was strongly and positively correlated with HTLV-1 mRNA load in CD4+ T cells of HAM/TSP patients (Pearson's *p*<0.0001) (Figure 2C).

Since high proviral load is a hallmark of HAM/TSP pathology [46], we investigated the relationship between HTLV-1 replication and *SOCS1* gene expression. Initially, the level of SOCS1 mRNA was examined in HTLV-1-carrying T cell lines (MT-2, C8166, MT-4, RMP), control T cell lines (Jurkat and CEM, Figure 2D and Figure S2), as well as PBMCs infected with the HTLV-1 infectious molecular clone pX1M-TM (Figure 2D and 2E). In non-leukemic MT-2 cells that carry an integrated replication-competent provirus and produce infectious HTLV-1 viral particles, a ∼50-fold increase in SOCS1 mRNA was detected, as compared to non-infected CEM and Jurkat cells (<5-fold). In leukemic MT-4 and C8166 cells, which carry a defective provirus, lower levels of SOCS1 mRNA were detected, suggesting that SOCS1 induction required an intact proviral genome (Figure 2D). The RMP cell line derived from an ATL patient which express low amount of HTLV-1 mRNA also displayed lower SOCS1 level (∼10-fold). In order to determine whether *de novo* HTLV-1 infection induced SOCS1 expression, PBMCs expressing the HTLV-1 infectious molecular clone pX1M-TM (Figure 2E) were analyzed for the level of SOCS1 and HTLV-1 mRNA at different times post-transfection. HTLV-1 RNA expression was determined by amplifying the pX region (*tax*/*rex*) of the HTLV-1 proviral genome; HTLV-1 RNA expression was modest between 24 and 72 h (<10-fold), but the viral mRNA load increased sharply at 96 h (50-fold), concomitent with a dramatic increase in *SOCS1* gene expression (∼24-fold).

**Figure 3:**
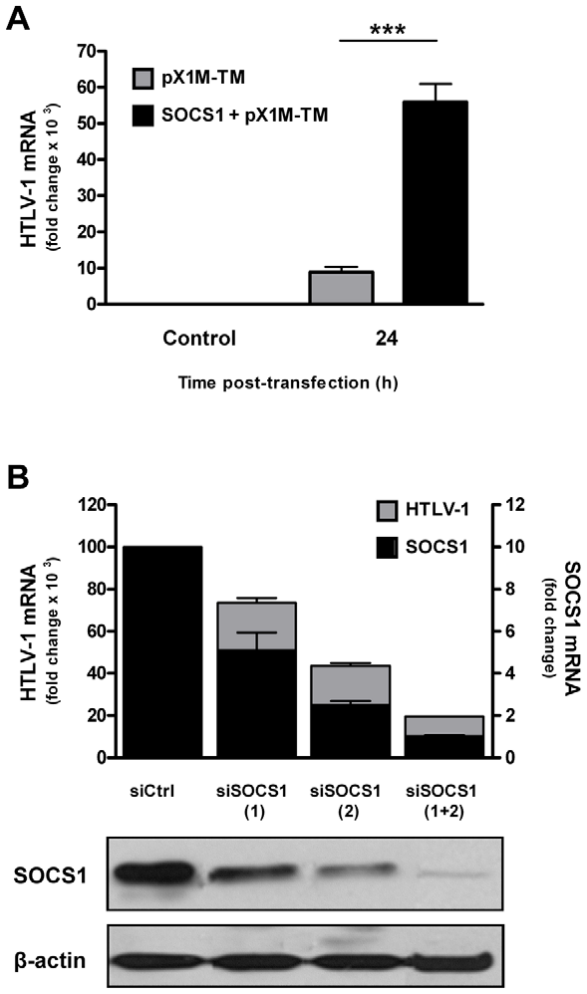
SOCS1 promotes HTLV-1 mRNA synthesis. (A) CEM cells were transfected with pX1M-TM alone or co-transfected with pX1M-TM and Myc-tagged SOCS1 expression vectors. At 24 h post-transfection, total RNA was extracted and evaluated for HTLV-1 (A) gene expression by real time PCR. Equivalent mRNA amounts were normalized to GAPDH mRNA expression and calculated as fold change of the levels of control cells which were arbitrarily set as 1 (*** *p*<0.001). (B) Depletion of SOCS1 decreases HTLV-1 mRNA synthesis in MT-2 cells. MT-2 cells were electroporated with control or SOCS1 specific-siRNAs (siSOCS1 (1), siSOCS1 (2), or a pool of both siSOCS1 (1) and siSOCS (2)). At 72 h post-transfection, total RNA was extracted and analyzed for HTLV-1 and SOCS1 mRNA levels. MT2 cells were treated as in B; cells lysates were prepared at 72 h post-electroporation, and equal amounts of protein (20 µg) were resolved by SDS-PAGE followed by immunoblotting against SOCS1, with β-actin shown as a loading control (bottom panel).

The initial observation that SOCS1 was induced upon HTLV-1 infection prompted us to examine whether SOCS1 also influenced viral replication. To do so, the effect of SOCS1 expression on HTLV-1 provirus replication was examined in the CEM T cells, co-expressing a SOCS1 expression vector together with the HTLV-1 provirus. The level of HTLV-1 mRNA was consistently higher in SOCS1 expressing cells compared to CEM cells expressing HTLV-1 provirus alone (e.g. 55-fold vs. 10-fold at 24 h) (Figure 3A). As a complementary strategy, SOCS1 expression was silenced in MT-2 cells (Figure 3B); siRNAs targeting SOCS1 (siSOCS1(1) siSOCS1(2) and siSOCS1(1)+siSOCS1(2)) inhibited SOCS1 levels by 50, 75 and 90%, respectively. Knock-down of SOCS1 protein expression was confirmed by immunoblot assay (Figure 3B, bottom panel). Real time PCR analysis of the HTLV-1 pX region demonstrated a significant reduction of HTLV-1 mRNA that directly correlated with the decrease in the observed SOCS1 levels (∼27, 56 and 80% decrease, respectively). These data indicate that SOCS1 induction during HTLV-1 infection leads to enhanced HTLV-1 replication.

### HTLV-1 suppresses type I IFN production

**Figure 4:**
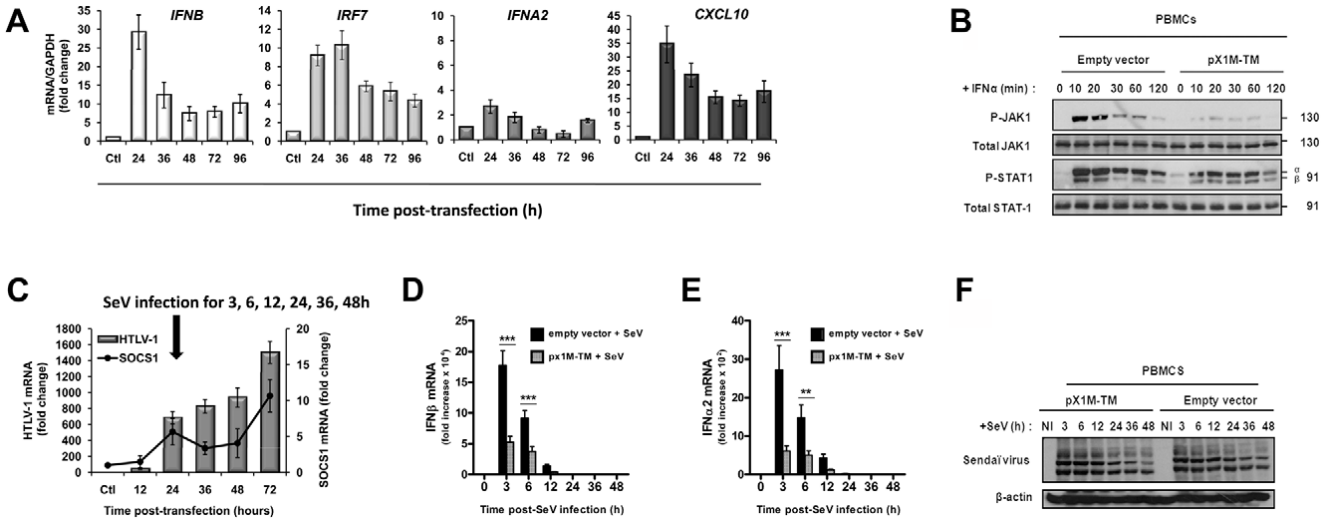
HTLV-1 infection results in the inhibition of type I IFN gene expression. (A) PBMCs from healthy individuals were electroporated with either HTLV-1 provirus (pX1M-TM) or control empty vector (ctl). At the indicated times, total RNA was extracted and analyzed for, *IFN-β*, *IRF7*, *IFN-α_2_*, *CXCL10* (A) gene expression. Equivalent mRNA amounts were normalized to *GAPDH* mRNA expression and calculated as fold change from the levels of control cells that were arbitrarily set as 1. (B) HTLV-1 inhibits IFN-α-induced tyrosine phosphorylation of JAK1 and STAT1. PBMCs were electroporated as in (A) and 48 h post-transfection cells were left-untreated or treated with 1000U/ml IFN-α. Cells lysates were prepared at indicated times and equal amounts of protein (50 µg) were resolved by SDS-PAGE followed by immunoblotting against Tyr-701-phosphorylated STAT1, total STAT1, Tyr-1022/1023-phosphorylated JAK1, total JAK1, with β-actin shown as a loading control. (C, D, E, F) HTLV-1 inhibits SeV-mediated type I IFN gene expression. PBMCs were electroporated with either HTLV-1 provirus (pX1M-TM) or control empty vector for 24 h prior to SeV infection. Total RNA was extracted at the indicated times and analyzed for HTLV-1 (C), IFN-β (D) and IFNα_2_ (E) mRNA levels by real time PCR. Equivalent mRNA amounts were normalized to GAPDH mRNA expression and calculated as fold change from the expression levels of control cells that were arbitrarily set as 1 (**, *p*<0.05; *** *p*<0.001). (F) PBMCs were treated as in C, D, E; cells lysates were prepared at 3–48 h post-SeV infection, and equal amounts of protein (20 µg) were resolved by SDS-PAGE followed by immunoblotting with anti-SeV antisera. Immunoblotting against β-actin is shown as a loading control.

Since SOCS1 has been shown to negatively regulate type I IFN signaling [34], [42], we sought to investigate the relationship between HTLV-1 infection, type I IFN response and SOCS1 gene expression. First, the profile of type I IFN (IFN-β and IFN-α_2_ *)* and IFN-stimulated gene expression (IRF7 and CXCL10) was examined in PBMCs expressing the HTLV-1 provirus pX1M-TM (Figure 4A). IFN-β, IFN-α_2_ and CXCL10 mRNAs were induced (30, 3.5 and 35-fold, respectively) at 24 h post-HTLV-1 transfection, and IRF7 mRNA expression (∼11-fold) was maximal at 36 h. However, mRNA transcripts for all these genes decreased substantially (below 50% of maximal levels) by 48–72 h. At 96 h, when HTLV-1 and *SOCS1* gene expression were maximal, no reactivation of antiviral gene transcription was detected (Fig 2D and 4A). We interpret this result as indicating that early after infection, transient stimulation of the antiviral response occurs and restricts *de novo* HTLV-1 RNA expression; at 72–96 h after infection induction of SOCS1 results in the shutdown of the type I IFN response, thus promoting high HTLV-1 mRNA expression.

IFN-α signaling is initiated by binding to the heterodimeric IFN-α receptor, followed by activation of JAK1 and TYK2 protein kinases, resulting in the phosphorylation of STAT1 and STAT2 [31]. To investigate whether HTLV-1 expression interfered with the type I IFN response, primary PBMCs expressing the HTLV-1 provirus pX1M-TM were treated with IFN-α for 10–120 min to focus on early IFN-triggered phosphorylation events. In control PBMCs, STAT1 and JAK1 phosphorylation was detected at 10 and 20 min post-IFN-α treatment, as determined by immunoblotting with specific antibodies (Figure 4B). However, in PBMCs expressing the proviral clone pX1M-TM, IFN-α-induced phosphorylation of JAK1 and STAT1 was reduced >90 and 70%, respectively (Figure 4B), while total protein levels of JAK1 and STAT1 remained unchanged in control and HTLV-1 expressing PBMCs.

To further characterize the effect of HTLV-1 on antiviral response, PBMCs expressing the HTLV-1 provirus were infected with Sendai virus (SeV) - a strong inducer of the antiviral response - and kinetics of expression of IFN genes was assessed by Q-PCR (Figure 4C, D, E). At 24 h post-transfection, PBMCs had significant HTLV-1 proviral load (∼700 fold higher than control, Figure 4C); thus at this time, PBMCs were infected with SeV (20 HAU/mL) to compare the levels IFN-α_2_ and IFN-β mRNA in the presence or absence of HTLV-1 provirus (Figure 4D and E). Induction of IFN-β and IFN-α_2_ mRNA was detected in all PBMCs as early as 3 h post-SeV infection and was sustained up to 12 h (Figure 4D and E). However, in HTLV-1 expressing PBMCs, induction of IFN-β and IFN-α_2_ mRNA was reduced >60%, relative to the level observed in the absence of HTLV-1 provirus. Decreased levels of IFN-β and IFN-α_2_ in cells expressing the HTLV-1 provirus were not due to inhibition of SeV replication, as demonstrated by immunoblot for SeV proteins (Figure 4F).

**Figure 5:**
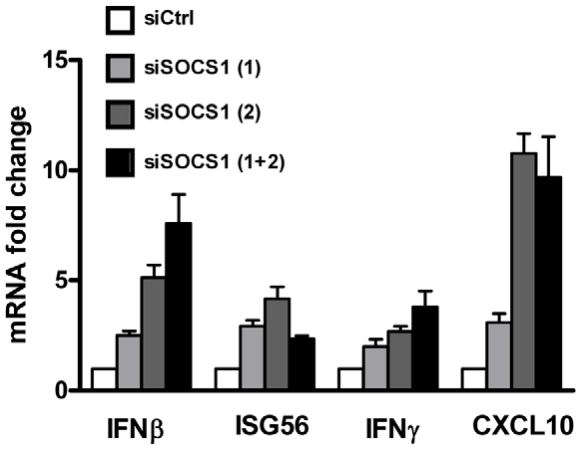
SOCS1 suppresses the antiviral response. Depletion of SOCS1 restores the type I and II IFN signaling in MT-2 cells. MT-2 cells were electroporated with control or SOCS1 specific-siRNAs (siSOCS1 (1), siSOCS1 (2), or a pool of siSOCS1 (1) and siSOCS1 (2)). At 72 h post-transfection, total RNA was extracted and analyzed for IFN-β, ISG56, CXCL10 and IFN-γ mRNA levels.

Similarly, knockdown of SOCS1 in MT-2 cells reversed the inhibition of antiviral gene expression imposed by HTLV-1 (Figure 5). Pooled siSOCS1 resulted in increased *IFN-*β (7.5-fold), *ISG56* (2.5-fold), *IFN-γ* (4-fold) and *CXCL10* (∼10-fold) gene expression compared to control siRNA. These results demonstrate that SOCS1 contributes to the inhibition of antiviral responses during HTLV-1 infection.

### HTLV-1-mediated SOCS1 expression induces proteasomal degradation of IRF3

**Figure 6:**
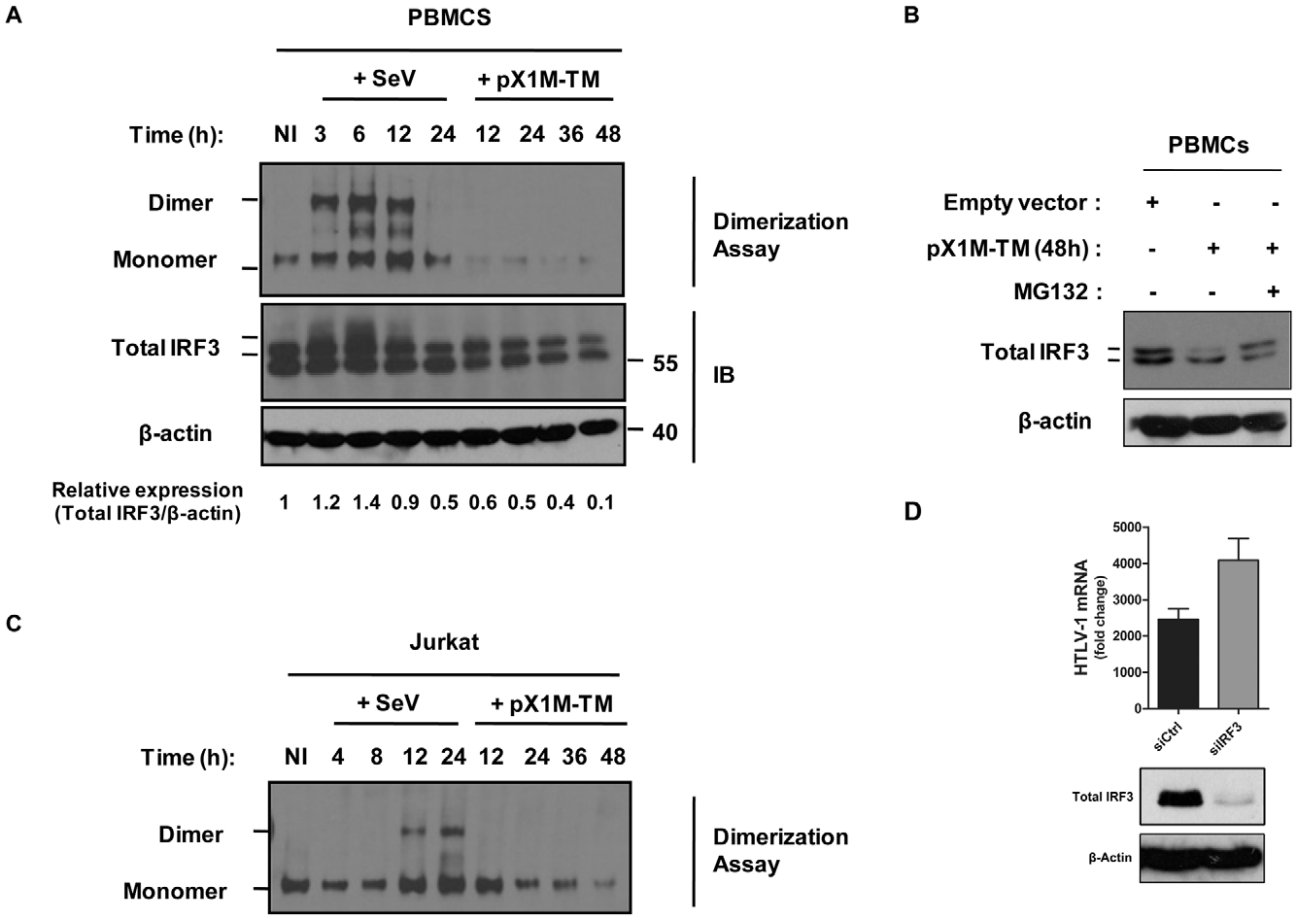
HTLV-1 induces degradation of endogenous IRF3. (A). PBMCs from healthy individuals were electroporated with either HTLV-1 provirus (pX1M-TM) or empty vector. PBMCs transfected with the control vector were subsequently infected with SeV. At the indicated times, lysates from transfected or infected PBMCs were electrophoretically resolved under non-denaturing or denaturing conditions. Western blot analysis was used to locate monomer and dimer forms of IRF3 in the non-denaturing gel (upper panel), and global IRF3 protein in the denaturing gel (lower panel). Immunoblotting against β-actin was used as a loading control. Total IRF3 protein expression levels (upper band) were quantified and normalized to β-actin levels using the Scion Image 4.0 software program. (B) Degradation of IRF3 is inhibited by the proteasome inhibitor (MG132). PBMCs were treated as indicated in (A). At 48 h post-HTLV-1 provirus, cells were incubated with 5 µM of MG132 for 6 h. Cells lysates were prepared and equal amounts of protein (20 µg) were resolved by SDS-PAGE followed by immunoblotting against IRF3, with β-actin shown as a loading control. (C) Same experiments and analysis for IRF3 dimerization were performed in the Jurkat leukemic T cell line. (D) Loss of IRF3 enhances HTLV-1 mRNA load in infected T cells. Jurkat cells were electroporated with control or a pool of IRF3 specific-siRNAs, and re-transfected at 24 h with HTLV-1 provirus (pX1M-TM) vector. At 72 h post-transfection, total RNA was extracted and analyzed for HTLV-1 mRNA levels. Cells lysates were prepared at 72 h post-electroporation, and equal amounts of protein (20 µg) were resolved by SDS-PAGE followed by immunoblotting against IRF3, with β-actin shown as a loading control.

Many pathogenic viruses strategically antagonize the early innate antiviral defenses in order to maintain viral replication, often inactivating IFN signaling components as part of their immune evasion strategy (reviewed in [45]). Because IRF3 is essential for IFN gene activation, we assessed IRF3 dimerization (as a measure of activation) in PBMCs expressing the HTLV-1 provirus (Figure 6A). In control PBMCs, SeV infection induced IRF3 dimer formation at 3–12 h post-infection, whereas IRF3 dimer formation was not detected in PBMCs expressing the HTLV-1 provirus. Furthermore, IRF3 monomer levels decreased sharply during the course of HTLV-1 replication (Figure 6A). Immunoblot analysis for total IRF3 confirmed that IRF3 levels decreased in a time dependent manner in PBMCs and Jurkat cells expressing the HTLV-1 provirus, a phenomenon not observed in control PBMCs infected with SeV (Figure 6C). IRF-3 was degraded *via* the proteasomal pathway, as the use of the proteasome inhibitor MG132 prevented HTLV-1 mediated reduction of IRF3 protein level (Figure 6B). This observation demonstrates for the first time that HTLV-1 does not activate IRF3 in PBMCs, but rather prevents the initial steps of type I IFN production by targeting IRF3 for proteasomal degradation. Additionally, IRF3 silencing in Jurkat cells expressing the HTLV-1 provirus resulted in increased HTLV-1 mRNA expression (Figure 6D) – indicating that the degradation of IRF3 by SOCS1 enhances viral mRNA load.

**Figure 7:**
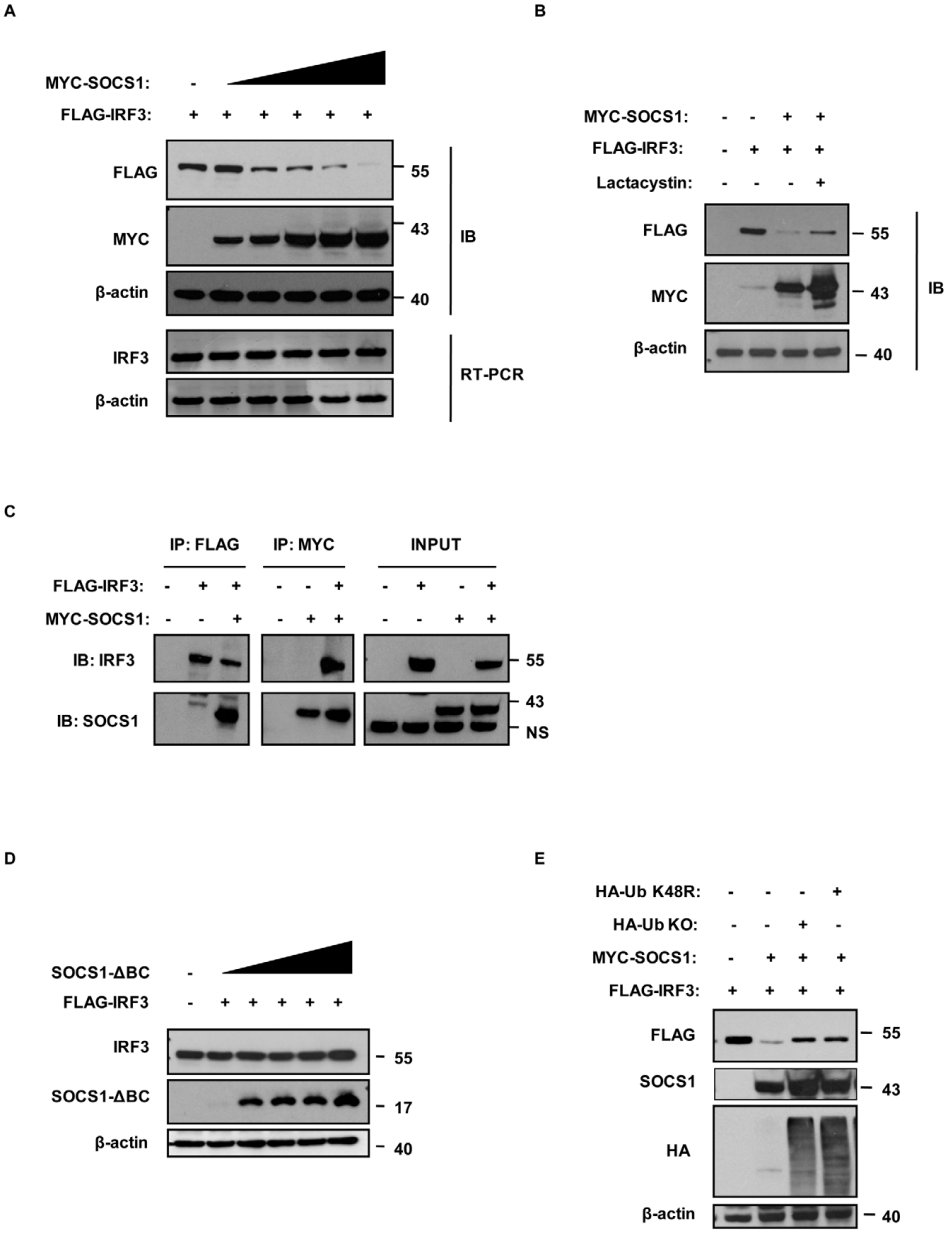
IRF3 interacts with SOCS1 and undergoes SOCS1-mediated degradation and ubiquitination. (A) HEK293 T cells co-transfected with increasing amounts of SOCS1 promotes degradation of IRF3 in a concentration-dependent manner. HEK293 T cells were co-transfected with Flag-tagged IRF3 and increasing amount of Myc-tagged SOCS1 expression vectors. Cells were collected 24 h post-transfection for either RNA extraction or whole cell extract (WCE) preparation. Immunoblot analysis for SOCS1 and IRF3 protein expression was performed by incubating with antibodies against Myc, Flag, or β-actin, respectively (upper panel). RNA (1 µg) from each sample was subjected to RT-PCR for selective amplification of specific IRF3 mRNA and the constitutively expressed β-actin, as a control. PCR products were separated on a 2% agarose gel and visualized with ethidium bromide staining (lower panel). (B) Degradation of IRF3 is inhibited by the proteasome inhibitor (lactacytin). HEK293 T cells were co-transfected with expression vectors as indicated in (A) and incubated with 10 µM of lactacystin. Twenty four hours post-transfection equal amounts of proteins (30 µg) were resolved by SDS-PAGE followed by immunoblotting with antiserum specific for Flag and Myc. Immunoblotting against β-actin was performed as loading control. (C) Interaction between IRF3 and SOCS1. HEK293 T cells were co-transfected with expresssion vectors for Myc-tagged SOCS1 and Flag-tagged IRF3; at 24 h post-transfection, cells lysates were immunoprecipitated with Flag or Myc antibodies, and the precipitates were immunoblotted to assess Flag and Myc proteins expression. (D) IRF3 is degraded in a SOCS-Box-dependent manner. HEK293 T cells were co-transfected with Flag-tagged IRF3 and increasing amount of a deletion mutant of SOCS1 (SOCS1-ΔBC-Box) expression vectors. Cells were collected 24 h post-transfection for WCE preparation. Immunoblot analysis for SOCS1 and IRF3 protein expression were performed and incubated with antibodies against SOCS1, Flag, or β-actin, respectively. (E) SOCS1 mediated IRF3 ubiquitination. HEK293 T cells were co-transfected with Flag-tagged IRF3, Myc-tagged SOCS1 and either KO or K48R ubiquitin mutant expression vectors. Cells were collected 24 h post-transfection for WCE preparation. Immunoblot analysis for IRF3, SOCS1, Ub protein expression were performed and incubated with antibodies against Myc, Flag, Ha or β-actin, respectively.

Given that SOCS1 upregulation during HTLV-1 infection inhibits the expression of IFN and ISGs, we sought to investigate the role of SOCS1 in HTLV-1-mediated degradation of IRF3. In HEK293T cells expressing increasing amounts of SOCS1 together with a constant amount of IRF3, SOCS1 expression induced IRF3 degradation in a dose-dependent manner (Figure 7A). RT-PCR analysis with specific IRF3 primers showed that the level of IRF3 mRNA remained unchanged, indicating that SOCS1 had no effect on *IRF3* gene expression (Figure 7A). Moreover, SOCS1 silencing in HTLV-1 infected MT-2 cells restored endogenous IRF3 expression (Figure S3). Interestingly, the addition of the proteasome inhibitors lactacystin (Figure 7B) or MG132 (data not shown) prevented IRF3 degradation in the presence of SOCS1. Furthemore, co-immunoprecipitation experiments demonstrated that SOCS1 physically interacted with IRF3 (Figure 7C), indicaing that IRF3 degradation was triggered by physical association with SOCS1.

SOCS1 induces degradation of target proteins by recruiting Elongin B/C to its SOCS-Box domain, leading to the formation of an E3 ubiquitin ligase complex able to modify substrate proteins with K48-linked ubiquitin chains. To confirm that SOCS1-mediated IRF3 degradation required E3 ligase complex activity, increasing amounts of a SOCS1 deletion mutant lacking the SOCS-Box - SOCS1-ΔB/C-Box - was expressed together with a constant amount of IRF3 (Figure 7D); SOCS1-ΔBC did not induce IRF3 degradation at any concentration (compare Figures 7D and 7A).

Proteasome-mediated degradation requires the addition of K48-polyubiquitin chain to the target protein; exogenous addition of ubiquitin mutated in its ability to link K48-polyubiquitin chains (ubiquitin-K48R, which contains a single K48R point mutation, or Ubi-KO, which contains no lysines) prevented IRF-3degradation, while HEK293 cells expressing exogenous SOCS1 readily degraded IRF3 (Figure 7E). In addition, IRF3 turnover was completely reversed in the presence of a 10-fold excess of HA-Ub K48R or HA-Ub KO (Figure 7E), thus confirming that proteosome-mediated IRF3 degradation by SOCS1 requires recruitment of Elongin B/C E3 ligase machinery and is dependent on K48-polyubiquitin chain formation.

## Discussion

The complexity of gene expression dysregulation in ATL or HAM/TSP diseases has been highlighted in a number of gene expression profiling [47], [48] and protein profiling studies [49]. The present study however represents the first comparative genome-wide array analysis to establish gene expression profiles for HTLV-1-associated disease states. With a unique cohort of 30 HTLV-1-infected individuals from the Caribbean basin and a custom ImmuneArray [50], we identified ∼1039 significant immune-related genes that were differentially regulated in CD4+ T cells from 11 AC, 7 ATL, 12 HAM/TSP patients, compared with CD4+ T cells from 8 NI donors from the same geographical region. Clear clinical discrimination was observed between the ATL, HAM/TSP and NI patients, both by unsupervised hierarchical cluster and principal component analysis. Our analysis revealed that the gene expression profile in ATL cells was clearly distinct from healthy CD4+ T cells, although similarities in gene expression patterns were observed between HAM/TSP samples and NI controls. This difference between HAM/TSP and ATL CD4+ T cells likely reflects numerous alterations in gene expression that occur during ATL transformation [12]. In contrast, evolution to HAM/TSP does not involve cellular transformation, but rather is characterized by a high HTLV-1 proviral load and the establishment of a pro-inflammatory microenvironment due to cytokine/chemokine production of infected and bystander immune cells. It is possible that T lymphocytes derived from early-stage HAM/TSP patients have a profile similar to healthy cells and that gene expression changes are observed only at later stages of the disease, an interesting hypothesis that needs to be investigated further. Interestingly, AC patients did not cluster as an individual group but rather distributed amongst NI, ATL and HAM/TSP patients, suggesting that extensive analysis of the genes modulated in NI, HAM/TSP and/or ATL groups may help to identify candidate genes important for early diagnosis of HTLV-1 diseases. Here, the major cellular pathways identified involved cell adhesion (CXCR4, CD2, CD63), antimicrobial defense (KLRB1, SPN, SELPLG), innate immune signaling (SOCS1, TRAF3, AIM2, TLR2, IKBKG, STAT3), antigen presentation (TRA alpha locus), and chemotaxis (CCL14, SPN, CCL13) thus supporting the idea of a global disruption of the immune system during HTLV-1 infection.

Among the many genes modulated during HTLV-1 infection, the suppressor of the interferon signaling - SOCS1 - was upregulated in HAM and AC patients but not in ATL. This observation is in agreement with a previous report published by Nishiura *et al.* demonstrating that SOCS1 mRNA levels were increased in HAM/TSP patients compared to NI [44]. We now demonstrate that CD4+ T cells from HAM/TSP and AC patients express increased levels of SOCS1 which strongly correlates with HTLV-1 mRNA load. Since HAM/TSP patients are characterized by a very high proviral load, we hypothesized that SOCS1 upregulation in HAM/TSP may represent an immune evasion strategy used by HTLV-1 to dampen the early IFN antiviral response. Indeed, in PBMCs expressing a HTLV-1 infectious molecular clone, and in cell lines harboring an intact HTLV-1 provirus, high levels of *SOCS1* gene expression correlated with high levels of HTLV-1 transcription. Increasing HTLV-1 proviral expression blocked expression of type I IFN genes such as IFN-β, IRF7, IFN-α_2_, as well as the IFN-γ stimulated chemokine gene CXCL10, with maximal inhibition observed when HTLV-1 and *SOCS1* gene expression levels were coordinately elevated. Furthermore, depletion of SOCS1 using siRNA decreased HTLV-1 replication and restored the type I IFN response.

IFN-α/β is known to have a negative impact on retrovirus replication. Although few studies have reported its effect on HTLV-1, type I IFN constitutes a potent anti-retroviral mechanism that limits HTLV-1 replication [22], [23]. Moreover, clinical studies using IFN-β therapy in HAM/TSP patients have demonstrated benefits in reducing HTLV-1 mRNA load and the number of pathogenic CD8+ T cells, as well as minimizing disease progression during therapy [51]. Accumulating evidence indicates that HTLV-1 possesses evasion mechanisms to counteract type I IFN signaling: for example, HTLV-1 down-regulates JAK-STAT activation by reducing phosphorylation of Tyk2 and STAT2, possibly through a Gag- or Pr-mediated mechanism [52]; and Tax further negatively modulates IFN-α-induced JAK/STAT signaling by competing with STAT2 for CBP/p300 coactivators [53].

SOCS1 is a cytokine-inducible intracellular negative regulator that inhibits type I and II IFN signaling by triggering the degradation of various components of the JAK-STAT cascade (reviewed in [32], [54]). SOCS1 can also be induced during virus infection and plays a positive role in viral replication [55], [56], [57]. SOCS1 is induced during virus infection and binds directly to the type I IFN and/or II IFN receptors to suppress IFN signaling, thereby preventing chronic inflammation. However, SOCS1 could be subverted to enhance viral replication via untimely inhibition of the IFN response. SOCS1 induction may be a direct result of viral protein activity. Bioinformatics analysis of the SOCS1 promoter region reveal the presence of CRE, AP-1 and NF-κB binding regions, suggesting the possible involvement of HTLV-1 Tax in the induction of SOCS1 expression (data not shown). Another possibility is that SOCS1 transcriptional activation is not directly regulated by viral proteins, but rather by recognition of viral RNA and downstream signaling events. For instance, Potlichet *et al.* reported that Influenza A virus suppresses the antiviral response by inducing SOCS1 and SOCS3 via TLR3-independent but RIG-I/IFNAR dependent pathways [43]. Moreover, IFN-γ gene expression in CD4+ T cells from HAM/TSP patients is elevated as compared to ATL or AC patients. Constitutive induction of IFN-γ may also augment SOCS1 expression, and thus increase HTLV-1 replication.

SOCS proteins exert their negative effect by promoting the ubiquitination and proteosomal degradation of key proteins involved in cytokine signaling pathways: MAL in Toll like receptor 4 signaling (TLR4), JAK2 in IFN-γ mediated signaling and NF-κB p65/RelA are all known targets of SOCS1 [38], [58], [59]. Here, we identified IRF3 as an important target for SOCS1-induced proteasomal degradation that impacts the early type I IFN antiviral response. IRF3 is ubiquitously expressed in the cytoplasm and is activated in response to viral infection, triggering IFN-β and other early ISGs expression, thus initiating the antiviral response. To counter type I IFN, many viruses have evolved strategies to interfere with IRF3 activation as an efficient means to limit IFN-β production [26], [60]. Interference of IRF3 activation also dampens the second wave of IFN signaling, including production of IFN-α. The mechanisms of IRF3 antagonism vary, and include inhibition of IRF3 phosphorylation, nuclear translocation, or transcription complex assembly as well as down-regulation of IRF3 by ubiquitin-mediated degradation. In this context, bovine herpes virus 1 infected cell protein 0 (bICP0) has been shown to act as an E3 ligase and promote IRF3 degradation in a proteasome-dependent manner, thus inhibiting the IFN response [61].

The interaction between SOCS1 and IRF3 during HTLV-1 infection promotes proteasome-mediated degradation of IRF3 and thus abrogates early IFN antiviral signaling. SOCS1-dependent IRF3 degradation required the elongin B and C binding sites within SOCS1 and K48-linked polyubiquitination of IRF3. Indeed, the SOCS box-mediated function of SOCS1 is chiefly exerted via its ubiquitin ligase activity [62] and biochemical binding studies have shown that the SOCS box interacts with the elongin B/C complex, a component of the ubiquitin/proteasome pathway that forms an E3 ligase with Cul2 (or Cul5) and Rbx-1 [40], [58]. Thus, SOCS1 serves as an adaptor to bring target proteins to the elongin B/C-Cullin E3 ligase complex for ubiquitination. Although we show from our current experiments that SOCS1 directly mediates K48-linked ubiquitination of IRF3, further studies are required to elucidate the details of SOCS1-mediated IRF3 ubiquitination, as well as the mechanisms of regulation of SOCS1 during HTLV-1 infection.

The present study reveals a novel mechanism of viral evasion of the IFN response in HTLV-1 infected T lymphocytes – the consequence of which can be directly related to the efficiency of HTLV-1 replication in patients suffering from HAM/TSP. Future studies are required to elucidate putative alternate consequences of SOCS1 upregulation in T cells [63], as well as the effect of HTLV-1 induced SOCS1 expression in other relevant viral reservoirs such as dendritic cells and astrocytes [64], [65]. Collectively, SOCS1-mediated degradation of IRF3 during HTLV-1 infection has substantial implications in the framework of known HTLV-1 pathobiology and as such opens new avenues of exploration for designing effective therapeutic strategies.

## Materials and Methods

### Ethics statement

Blood samples from HTLV-1 infected patients and non-infected (NI) donors were obtained from the Centre Hospitalier Universitaire de Fort-de-France in Martinique and Institut Pasteur de Cayenne in French Guyana. Patients suffering from ATL, HAM/TSP or HTLV-1 asymptomatic carriers were recruited according to World Health Organization (WHO) criteria. According to the French Bioethics laws, the collection of samples from HAM/TSP, ATL, AC and NI has been declared to the French Ministry of Research and the study was reviewed and approved by the CPP (Comité de Protection des Personnes) Sud-Ouest/Outre-Mer III, as well as the ARH (Agence Régionale de l'Hospitalisation) from Martinique. Because the protocol is non-interventional (e.g. blood samples collected for routine health care with no additional samplings or specific procedures for subjects), no informed consent was provided by the patient, as stated by the French Public Health code and therefore the study was conducted anonymously. Clinical collection of samples for research purpose are stored at the Centre de Ressources Biologiques de Martinique (CeRBiM). The CeRBiM database has been approved by the CNIL (Commission nationale de l'informatique et des libertés). Leukophoresis from healthy donors were also obtained at the Royal Victoria Hospital, Montreal, Quebec, Canada. Informed consent were written and provided by study participants in accordance with the Declaration of Helsinki. The study was reviewed and approved by the Royal Victoria Hospital, the Jewish General Hospital, and McGill University Research Ethics Committee (REC) board of the SMBD-Jewish General Hospital.

### Patient samples

In total, we selected for study 12 HAM/TSP, 11 asymptomatics (AC), 7 ATL and 8 not infected individuals (NI). The diagnosis of the 7 ATL cases included in patient cohort number 1 respected the international consensus recently published by Tsukasaki *et al*. [7]. Diagnostic criteria for ATL included serologic evidence of HTLV-1 infection, and cytologically or histologically proven T cell malignancy. Six ATL cases were classified as acute leukemia type on the basis of leukemic manifestations, with >5% typical ATL cells in the peripheral blood, and immunologically confirmed mature CD4+ T cell phenotype. One case (HISS0023) was a lymphoma type, with <5% circulating abnormal cells, the ATL cell phenotype and clonal integration of HTLV-1 being confirmed on lymph node tissue. Diagnosis of HAM/TSP was in accordance with WHO criteria [66], which comprise (1) slowly progressive spastic paraparesis with symmetrical pyramidal signs, (2) disturbance of bladder function, (3) no radiologic evidence of significant spinal cord compression, and (4) intra-thecal synthesis of anti−HTLV-1 antibodies. The asymptomatic HTLV-1 carriers did not display any neurological symptoms (Tables S1 and S2). PBMCs were isolated by centrifugation (400 g at 20°C for 25 min) on a Ficoll-Hypaque gradient (GE Healthcare Bio-Sciences Inc., Oakville, Canada). CD4+ T lymphocytes were isolated using a negative selection CD4 enrichment cocktail with the high-speed autoMACS system (Miltenyi Biotec) according to the manufacturer's instructions. In all cases, the purity of CD4+ T lymphocytes was between 90 and 95% as determined by flow cytometry. Cells were pellet and kept at −80°C until all samples were ready for RNA extraction.

### Cell lines and reagents

The HTLV-1-carrying T cell lines MT-2, MT-4, C8166, RMP and the HTLV-1-negative T cell lines CEM and Jurkat were used for experiments. MT-2, MT-4 and C8166 cells are derived from umbilical cord blood lymphocytes after cocultivation with leukemic cells from ATL patients [67]. MT-2 cells are reported to have integrated at least fifteen copies/cell, including defective types, of HTLV-1 proviral DNA whereas C8166 cells have only one copy of proviral DNA integrated in the genome [68], [69]. The interleukin (IL-2)-independent RMP cell line is derived from CD4+ T cell of a patient with acute ATL. All cell lines were maintained in RPMI 1640 medium supplemented with 10% heat-inactivated fetal bovine serum, 100 U/ml penicillinG, and 100 µg/ml streptomycin. HEK293T cells were used for transient transfection and were maintained in Dulbecco's modified Eagle's medium (DMEM) supplemented with 10% heat-inactivated fetal bovine serum, 100U/ml penicillin G, and 100 µg/ml streptomycin.

For proteasome inhibitor treatment, MG132 (Sigma-Aldrich) or Lactacystin (Boston Biochem) were used at 5 and 10 µM, respectively. Sendai virus CANTELL strain (SeV) was obtained from Charles River Laboratory (North Franklin, CT). Cells were infected with SeV at 20 hemagglutinating units (HAU) per 10^6^ cells in serum-free medium supplemented with 10% heat-inactivated fetal bovine serum 2 h postinfection and harvested for whole cell extracts or RNA extraction at indicated times.

### Plasmids

The HTLV-1 proviral clone pX1M-TM was kind gift from Dr David Derse (National Cancer Institute-Frederick, Frederick, USA). Myc**-**tagged SOCS1 full length and the deletion mutant SOCS1-ΔBCBox (amino acids 174–183) were kind gifts from Dr. Ferbeyre Gerardo (Departement de Biochimie, Universite de Montreal, Canada). Ha-Ub-K48R and Ha-Ub-KO were kind gifts from Dr. Zhijan Chen (Department of Molecular Biology, University of Texas Southwestern Medical Center, Dallas, Texas). Plasmid encoding for Flag-tagged IRF3 full length was decribed previously [27].

### RNA isolation, amplification, and hybridization

Total RNA was extracted using Trizol Reagent (Invitrogen) or RNeasy kit (Qiagen) according to the manufacturer's instructions. The RNA integrity and purity was assessed with the Agilent 2100 Bioanalyzer (Agilent Technologies). Total RNA was amplified using the MessageAmp II mRNA kit (Ambion, Austin, USA). Sample and universal human RNA probes (Stratagene) for microarray hybridization were prepared by labeling the amplified RNA with Cy5 or Cy3, respectively, by reverse transcription, and hybridizing the labeled cDNA on the CANVAC (http://www.canvac.ca/) human Immunoarray version 2 manufactured by the Microarray Center (UHN, Toronto, ON, Canada) containing 7256 duplicate spots representing 3628 expressed sequence tags (ESTs). Details of the labeling and hybridization procedures can be obtained at http://transnet.uhnres.utoronto.ca.

### Microarray analysis

Microarrays were scanned using Scanarray Express Scanner (Packard Biosciences) or the Axon 4000B scanner at 10-µm resolution. Array images were inspected visually for poor quality spots and flagged for omission. Quantified raw data was acquired with QuantArray version 3 and saved as quantarray text files. The quantified raw data were managed and pre-processed in GeneTraffic (Iobion Informatic). Following background correction and removal of genes where both channels were less than 100 or represented by less than 90% of the samples and polished data was generated by normalization by Lowess sub-grid. The final data array was analyzed using JExpress Pro software (http://www.molmine). To establish differentially expressed genes, multi-class analysis was performed by one-way ANOVA on Log_2_ fold change (Log_2_Fc) data for ATL, HAM/TSP, AC and NI groups. Genes with a *p* value ≤0.01 were selected as significant (1039 total). Visualization was produced by unsupervised clustering of the 1039 genes using Pearson correlation parameters. Pair wise correspondance analysis (PCA) was performed on the first 500 genes by Future Subset Selection (FSS) t-test. Genes were selected based on false discovery rate (FDR) according to the Benjamini/Hochberg (BH) methods. Gene annotations were gathered using manual searches in NCBI as well as the ontology tools DAVID (http://david.abcc.ncifcrf.gov/) and BioRag (Bioresource for array genes, http://www.biorag.org). Fold change (Fc) for each gene was calculated as 2^(Log^_2_^X-Log^_2_^NI)^, where Log_2_ X represents the Log_2_ (Fc) for either ATL, AC or HAM and Log_2_ NI represents the Log_2_ (Fc) for NI. Microarray data have been deposited in the NCBI Gene Expression Omnibus (http://www.ncbi.nlm.nih.gov/geo/). The results of the microarray experiment were confirmed by quantitative PCR (Q-PCR) on 47 genes chosen on the basis of a fold change of at least 2-fold, with RNA from 3 patients per group used for validation. A strong correlation between the average fold-change determined by microarray and the average of the qPCR results was observed, with 25/47 genes having a Pearson correlation value of at least 0.6 (Figure S1A, B, C) and 18/47 genes with a value of at least 0.9 (Figure S1A).

### Transfection of primary PBMCs with HTLV-1 proviral clone

Leukophoresis from healthy donors were obtained at the Royal Victoria Hospital, Montreal, Quebec, Canada. PBMCs were isolated by Ficoll-Hypaque gradient (GE Healthcare Bio-Sciences Inc., Oakville, Ontario, Canada) and activated for 4 days with 2 µg/ml of phytohemagglutinin-P (PHA-P) (Sigma Aldrich) and 50 U of interleukin 2 per ml (IL-2) (PBL Biomedical Laboratories). 5 µg of pX1M-TM was pulsed into 10×10^6^ cells PBMCs in a 0.4-cm cuvette using a Gene Pulser II (Bio-Rad Laboratories) set at 0.25 kV and 0.95 µF. Cells were plated in six-well plates in complete medium and collected at indicated times for whole cell extracts or RNA extraction.

### Real time PCR analysis

Validation of selected target genes was performed by relative quantification PCR (RQ-PCR) in 9 samples (3 NI, 3 ATL, 3 HAM). A total of 2 µg of amplified RNA from uninfected and HTLV-1-infected samples was converted to cDNA using the High Capacity cDNA Archive Kit (Applied Biosystems, Foster City, CA) according to the manufacturer's protocol. cDNA was amplified using SyBR Green I PCR master mix (Roche Applied Science, Germany) or TaqMan Universal PCR Master Mix (Applied Biosystems, Foster City, CA, USA). Real-time PCR primers were designed using the primer3 website (primer3_www.cgiv. 0.2) and listed in Supporting information (Table S3). Some predesigned primers and probe sets from TaqMan (Applied Biosystems) were also used and listed in Table S3. Data were then collected using the AB 7500 Real-Time PCR System (Applied Biosystems, Foster City, CA) and analyzed by comparative C_T_ method using the SDS v1.3.1 Relative Quantification (RQ) Software where ddCT  =  dCT(Sample) – dCT (non-infected), dCT (Sample)  =  CT (Sample) - CT (GAPDH) and dCT (non-infected)  =  CT (non-infected) - CT (GAPDH).

### Immunoblot analysis and dimerization assay

Cells destined for immunoblotting were washed with PBS and lysed in lysis buffer (0.05% NP-40, 1% glycerol, 30 mM NaF, 40 mM β-glycerophosphate, 10 mM Na_3_VO_4_, 10 ng/ml of protease inhibitors cocktail (Sigma Aldrich, Oakville, Ontario, Canada). The protein concentration was determined by using the Bradford assay (Bio-Rad, Mississauga, Canada). Whole-cell extracts (30 µg) were resolved by sodium dodecyl sulfate-polyacrylamide gel electrophoresis (SDS-PAGE) in a 10%-acrylamide gel and transferred to a nitrocellulose membrane (Bio-Rad, Mississauga, Canada). Membranes were blocked in 5% nonfat dried milk in Tris-buffered saline (TBS) plus 0.1% Tween 20 for 1 h at room temperature. Membranes were then probed overnight with antibodies against Stat 1 phosphorylated (Tyr701) (1∶1000; Cell Signaling) and non-phosphorylated forms (p91) (1 µg/ml; Santa Cruz); phosphorylated Jak1 (Tyr 1022/1023) (1∶1000; Cell Signaling); total Jak 1 (1 µg/ml Santa Cruz); SeV (1∶10,000); SOCS1 (1 µg/ml; Zymed laboratories), IRF3 (1∶10,000; IBL, Japan) in 5% bovine serum albumin and PBS at 4°C. Incubation mixtures were washed in TBS-0.05% Tween 20 five times for a total of 25 min. Following washes, the membrane was incubated with peroxidase-conjugated goat anti-rabbit or anti-mouse antibody (KPL, Gaithersburg, MD) at a dilution of 1∶5,000 for 1 h at room temperature. Following the incubation with the secondary antibody, membranes were washed again (5 times, 5 min each) and then visualized with an enhanced chemiluminescence (detection system as recommended by the manufacturer (ECL; GE Healthcare Bio-Sciences Inc., Oakville, Ontario, Canada).

Native-PAGE was conducted as described [70]. Briefly, 10 g WCE in native sample buffer (62.5 mMTris-HCl, pH 6.8, 15% glycerol, and bromophenol blue) were resolved by electrophoresis on a 7.5% acrylamide gel (without SDS) pre-runned for 30 min at 40 mA using 25 mMTris and 192 mM glycine, pH 8.4, with and without 1% deoxycholate in the cathode and anode chamber, respectively. After transferred into nitrocellulose membrane, IRF3 monomers and dimers were detected by immunoblot using an IRF3 anti-NES antibody (1∶10, 000, IBL, Japan).

### Immunoprecipitation assay

HEK293 cells (1×10^6^ cells/60-mm dish) were transiently transfected with equal amounts (5 µg) of IRF3 and MYC-tagged SOCS1 expression plasmids by using calcium phosphate precipitation method. Cells were harvested 24 h post-transfection, washed with 1 X phosphate-buffered saline (PBS), and lysed in a 1% Triton X-100 lysis buffer (20 mM Tris-HCl, pH 7.5, 200 mM NaCl, 1% Triton X-100, 10% glycerol, 40 mM β-glycerophosphate, 0.1% protease inhibitor cocktail, 1 mM phenylmethylsulfonyl fluoride, 1 mM Na_3_VO_4_, 5 mM NaF, 1 mM dithiothreitol). Immunoprecipitations were performed by incubating WCE (300 µg) with 1 µg of anti-MYC (9E10; Sigma-Aldrich, St. Louis, MO) or 1 µg of antiserum directed against IRF3 (rabbit polyclonal antibody, IBL, Japan) coupled to 50 µl of A/G Plus-agarose beads (Santa Cruz Biotechnology, Santa Cruz, CA) overnight at 4°C with constant agitation. Immunocomplexes were washed at least 3 times in lysis buffer eluted by boiling beads in 5 volumes SDS-PAGE sample buffer. The proteins were fractioned on 10% SDS-PAGE, transferred to nitrocellulose membrane and analyzed by immunoblot assay using anti-MYC (Sigma-Aldrich) or anti-IRF3 (IBL, Japan) antibodies.

### RNA interference

Control and SOCS1-specific RNA interference sequences were described previously [71], [72]. SOCS1 protein was knocked down using siSOCS (1), siSOCS (2) or a pool of the two siRNAs (siSOCS (1) + siSOCS (2)). siRNAs were pulsed into MT-2 cells in a 0.4-cm cuvette using a Gene Pulser II (Bio-Rad Laboratories) set at 0.25 kV and 0.95 µF. Cells were plated in six-well plates in complete medium, washed 4 and 12 h later and collected at 72 h post-transfection. RNA extinction efficiency was demonstrated by real time PCR and immunoblot assay.

### Statistical analysis

Data are presented as the mean ± standard error of the mean (SEM). Statistical significance for comparison of gene expression was assessed by an unpaired Student's *t* test, with the expection of Figure 4, panels D and E where a two-way ANOVA with Bonferroni post-test was used. Analyses were performed using Prism 5 software (GraphPad). Statistical significance was evaluated using the following *p* values: *p*<0.05 (*), *p*<0.01 (**) or *p*<0.001 (***).

### Accession numbers

A list of accession numbers for genes and proteins mention in this study are listed in Table S4.

## Supporting Information

Figure S1 Confirmation of microarray results by Q-PCR. (A) Total RNA of 9 donors (3 NI, 3 HAM, 3 ATL) was reverse transcribed and cDNA was amplified using primers specific for indicated genes by real-time PCR. Values were normalized to GAPDH and relative quantification (RQ) was calculated by the comparative CT method. (B) Raw data of array data (log_2_) and real-time PCR data (log_2_RQ) for select genes. (C) Pearson correlation data represent the strength and direction of the linear relationship between real-time PCR assays and microarray data represented in (B). Genes with a Pearson correlation value between 0.6 and 0.8 are represented in (C).
(0.95 MB TIF)

Figure S2 CEM and Jurkat CD4+ T cell lines express SOCS1 when treated with IFNα2b. Expression of SOCS1 was measured in HTLV-1-negative T cell lines (CEM, Jurkat) with and without 1000U/ml of IFNα2b. cDNA was analyzed by quantitative real time PCR to measured SOCS1 expression. Equivalent mRNA amounts were normalized to GAPDH expression and calculated as fold change with the levels of uninfected Jurkat cells set arbitrarily as 1.
(0.44 MB TIF)

Figure S3 SOCS1 silencing in HTLV-1 infected MT-2 cells restores endogenous IRF3 expression. MT-2 cells were electroporated with control or pool of SOCS1 specific-siRNAs. At 72 h post-transfection, cells lysates were prepared and equal amounts of protein (20 µg) were resolved by SDS-PAGE followed by immunoblotting with anti-SOCS1 or anti-IRF3 antibodies. Immunoblotting against β-actin is shown as a loading control.
(0.55 MB TIF)

Table S1 Cohort #1 of HTLV-1 infected and non-infected donors.
(0.07 MB DOC)

Table S2 Cohort #2 of HAM/TSP and non-infected donors.
(0.04 MB DOC)

Table S3 Supplemental material and methods for Q-PCR.
(0.03 MB DOC)

Table S4 Accession numbers.
(0.04 MB XLS)
