# Supplementary figures and images for: HTLV-1 Evades Type I Interferon Antiviral Signaling by Inducing the Suppressor of Cytokine Signaling 1 (SOCS1)
Source: PLoS Pathog. 2010 Nov 4;6(11):e1001177. doi: 10.1371/journal.ppat.1001177 (PMC2973829; doi:10.1371/journal.ppat.1001177)

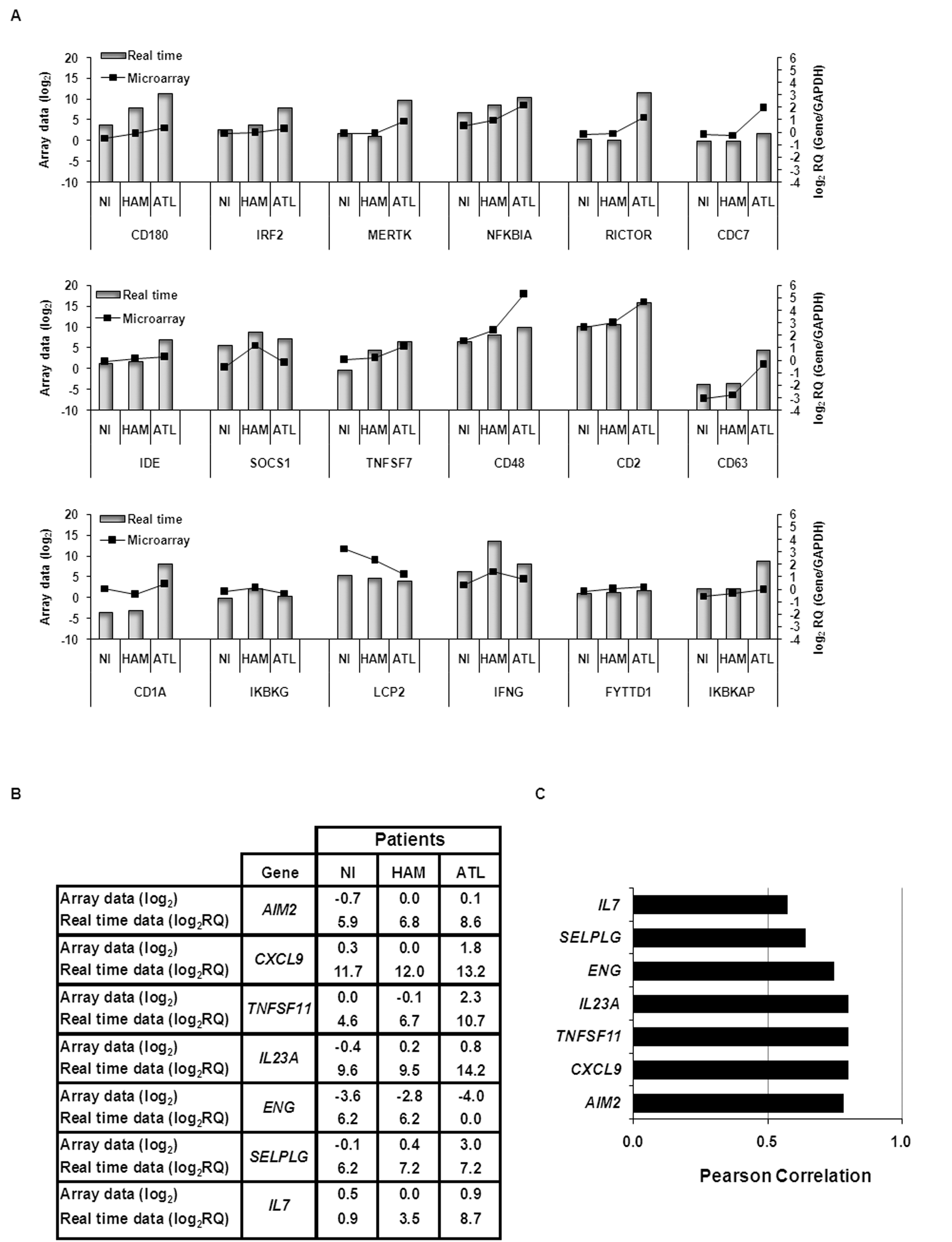

Supplement: Figure S1 — Confirmation of microarray results by Q-PCR. (A) Total RNA of 9 donors (3 NI, 3 HAM, 3 ATL) was reverse transcribed and cDNA was amplified using primers specific for indicated genes by real-time PCR. Values were normalized to GAPDH and relative quantification (RQ) was calculated by the comparative CT method. (B) Raw data of array data (log2) and real-time PCR data (log2RQ) for select genes. (C) Pearson correlation data represent the strength and direction of the linear relationship between real-time PCR assays and microarray data represented in (B). Genes with a Pearson correlation value between 0.6 and 0.8 are represented in (C). (0.95 MB TIF) [file ppat.1001177.s001.tif]

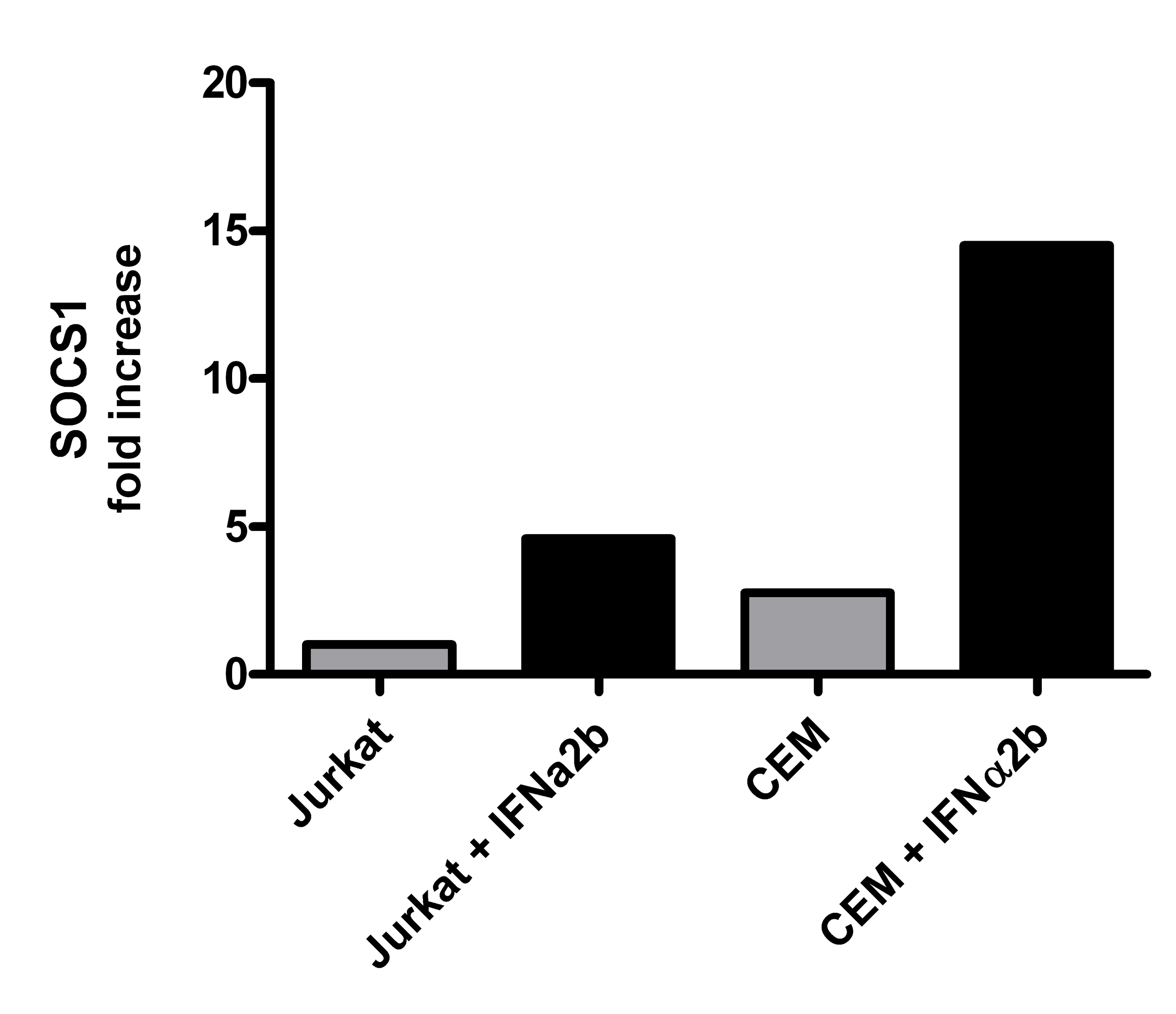

Supplement: Figure S2 — CEM and Jurkat CD4+ T cell lines express SOCS1 when treated with IFNα2b. Expression of SOCS1 was measured in HTLV-1-negative T cell lines (CEM, Jurkat) with and without 1000U/ml of IFNα2b. cDNA was analyzed by quantitative real time PCR to measured SOCS1 expression. Equivalent mRNA amounts were normalized to GAPDH expression and calculated as fold change with the levels of uninfected Jurkat cells set arbitrarily as 1. (0.44 MB TIF) [file ppat.1001177.s002.tif]

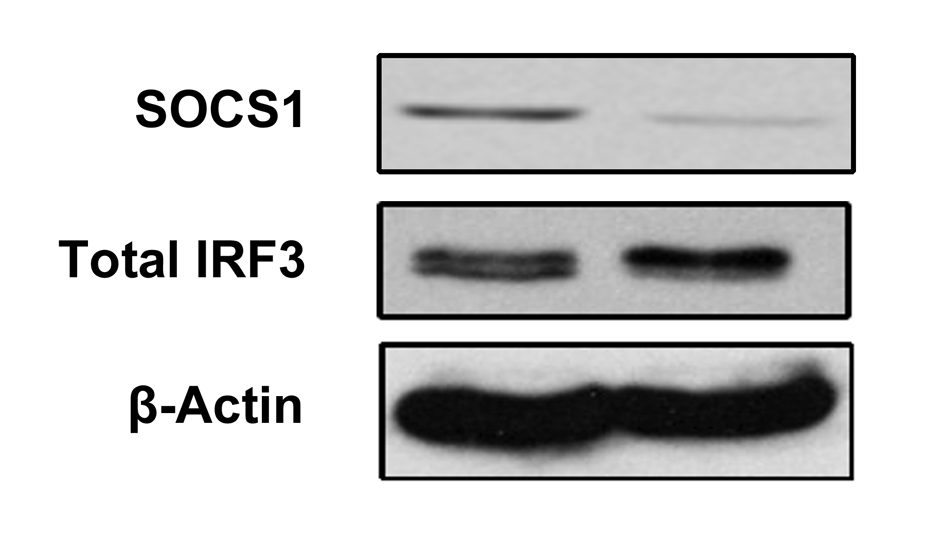

Supplement: Figure S3 — SOCS1 silencing in HTLV-1 infected MT-2 cells restores endogenous IRF3 expression. MT-2 cells were electroporated with control or pool of SOCS1 specific-siRNAs. At 72 h post-transfection, cells lysates were prepared and equal amounts of protein (20 µg) were resolved by SDS-PAGE followed by immunoblotting with anti-SOCS1 or anti-IRF3 antibodies. Immunoblotting against β-actin is shown as a loading control. (0.55 MB TIF) [file ppat.1001177.s003.tif]
