# Supplementary material for: HTLV-1 Evades Type I Interferon Antiviral Signaling by Inducing the Suppressor of Cytokine Signaling 1 (SOCS1)
Source: PLoS Pathog. 2010 Nov 4;6(11):e1001177. doi: 10.1371/journal.ppat.1001177 (PMC2973829; doi:10.1371/journal.ppat.1001177)
Supplement: Table S1 — Cohort #1 of HTLV-1 infected and non-infected donors. (0.07 MB DOC) [file ppat.1001177.s004.doc]

**Supplementary Table 1. Cohort #1 of HTLV-1 infected and non-infected donors***.* Patients recruited in Martinique and French Guyana for microarray study (Figure 1).

| **Hyb ID** | **Ethnicity** | **Gender** | **Diagnosis** | **Location** |  |
| --- | --- | --- | --- | --- | --- |
| HISS0042 | Black | M | AC | Martinique |  |
| HISS0044 | Black | F | AC | Martinique |  |
| HISS0045 | Black | F | AC | Martinique |  |
| HISS0046 | Black | F | AC | Martinique |  |
| HISS0047 | Black | M | AC | Martinique |  |
| HISS0013 | Black | F | AC | Guyana |  |
| HISS0014 | Black | F | AC | Guyana |  |
| HISS0015 | Black | M | AC | Guyana |  |
| HISS0056 | Black | ND | AC | Guyana |  |
| HISS0057 | Black | ND | AC | Guyana |  |
| HISS0058 | Black | ND | AC | Guyana |  |
|  |  |  |  |  | **ATL cells (%)** |
| HISS0017 | Black | ND | ATL | Martinique | 40% |
| HISS0018 | Black | F | ATL | Martinique | >5% |
| HISS0029 | Black | ND | ATL | Martinique | >5% |
| HISS0024 | Black | F | ATL | Guyana | 39% |
| HISS0010 | Black | F | ATL | Guyana | >5% |
| HISS0022 | Black | M | ATL | Guyana | 88% |
| HISS0023 | Black | M | ATL | Martinique | 3% |
|  |  |  |  |  | **EDSS** |
| HISS0021 | Black | F | HAM/TSP | Martinique | 5 |
| HISS0031 | Black | F | HAM/TSP | Martinique | 7 |
| HISS0040 | Black | F | HAM/TSP | Martinique | 6 |
| HISS0041 | Black | M | HAM/TSP | Martinique | 7.5 |
| HISS0043 | Black | M | HAM/TSP | Martinique | 6 |
| HISS0048 | Black | M | HAM/TSP | Martinique | 7.5 |
| HISS0049 | Black | M | HAM/TSP | Martinique | 6 |
| HISS0051 | Black | ND | HAM/TSP | Guyana | ND |
| HISS0052 | Black | ND | HAM/TSP | Guyana | ND |
| HISS0053 | Black | ND | HAM/TSP | Guyana | ND |
| HISS0054 | Black | ND | HAM/TSP | Guyana | ND |
| HISS0055 | Black | ND | HAM/TSP | Guyana | ND |
|  |  |  |  |  |  |
| HISS0060 | Black | ND | Healthy | Martinique |  |
| HISS0061 | Black | ND | Healthy | Martinique |  |
| HISS0062 | Black | ND | Healthy | Martinique |  |
| HISS0063 | Black | ND | Healthy | Martinique |  |
| HISS0064 | Black | ND | Healthy | Martinique |  |
| HISS0065 | Black | ND | Healthy | Martinique |  |
| HISS0066 | Black | ND | Healthy | Martinique |  |
| HISS0067 | Black | ND | Healthy | Martinique |  |

Hyb ID: microarray hybridization identifier; EDSS: Kurtzke Expanded Disability Status Scale; ND: not determined.
