# Supplementary material for: HTLV-1 Evades Type I Interferon Antiviral Signaling by Inducing the Suppressor of Cytokine Signaling 1 (SOCS1)
Source: PLoS Pathog. 2010 Nov 4;6(11):e1001177. doi: 10.1371/journal.ppat.1001177 (PMC2973829; doi:10.1371/journal.ppat.1001177)
Supplement: Table S2 — Cohort #2 of HAM/TSP and non-infected donors. (0.04 MB DOC) [file ppat.1001177.s005.doc]

**Supplementary Table 2. Cohort #2 of HAM/TSP and non-infected donors.** Patient samples used to validate increased SOCS1 expression and correlation with HTLV-1 proviral load for Figure 2C.

|  |  |  |  |  |  |
| --- | --- | --- | --- | --- | --- |
| **Patient ID** | **Ethnicity** | **Gender** | **Age** | **EDSS** | **Disease duration (in years)** |
|  |  |  |  |  |  |
| P1 | Black/African American | M | 72 | 6.0 | 13 |
| P2 | Black/African American | F | 51 | 6.0 | 6 |
| P3 | Black/African American | F | 54 | 8.0 | 3 |
| P4 | Caucasian | F | 69 | 2.5 | 11 |
| P5 | Black/African American | F | 54 | 6.5 | 2 |
| P6 | Hispanic | F | 43 | 6.0 | 5 |
| P7 | Black/African American | F | 47 | 6.0 | 8 |
| P8 | Black/African American | F | 61 | 6.5 | 14 |
| P9 | Black/African American | F | 68 | 6.5 | 12 |
| P10 | Black/African American | M | 64 | 1.0 | 22 |
|  |  |  |  |  |  |
| C694 |  |  |  | NA | NA |
| C750 |  |  |  | NA | NA |
| C185 |  |  |  | NA | NA |
| C211 |  |  |  | NA | NA |
| C125 |  |  |  | NA | NA |

EDSS: Kurtzke Expanded Disability Status Scale; NA: not applicable.
