# Supplementary material for: HTLV-1 Evades Type I Interferon Antiviral Signaling by Inducing the Suppressor of Cytokine Signaling 1 (SOCS1)
Source: PLoS Pathog. 2010 Nov 4;6(11):e1001177. doi: 10.1371/journal.ppat.1001177 (PMC2973829; doi:10.1371/journal.ppat.1001177)
Supplement: Table S3 — Supplemental material and methods for Q-PCR. (0.03 MB DOC) [file ppat.1001177.s006.doc]

**Table S3. Supplemental material and methods for Q-PCR.**

| **List of human primers used (SYBR GREEN)** |
| --- |

*NFKBIA*  F 5’-GATCACCAACCAGCCAGAAATT-3’

R 5’-TCTCGGAGCTCAGGATCACA-3

*RICTOR*  F 5’-CACTTACTACTTACCGGAAGCCTGTT-3’

R 5’-GGTAGACGTGAGGACGCTGTAAT-3’

*IRF2* F 5’-TCCTATGCAGAAAGCGAAACG-3’

R 5’-CCCCTCGGCACTCTCTTCA-3’

*CD180* F 5’-GAGGAGACCACGTGTGCAAA-3’

R 5’-CAGGAAAGCTTGACATCAGATAGC-3’

*CD2* F 5’-GAAATGATGAGGAGCTGGAGACA-3’

R 5’-CCGGCCCCTTTCTTCAGT-3’

*CD63* F 5’-GAGAATTACCCGAAAAACAACCA-3’

R 5’-CACTTAAAATCTGCCTGCATCCT-3’

*MERTK* F 5’-CGAGCTCGGATCTCTGTTCAA-3’

R 5’-GGTGACGGCTGCAATCCT-3’

*CD1A*  F 5’-TGACACCTGCCCACGTTTC-3’

R 5’-CCGCTGGAGATGTGCCTTT-3’

*SELPLG* F 5’-GCCTGCTGGCCATCCTAA-3’

R 3’-ACCGCCAGCACCACAGT-3’

*CXCL10* F 5’-TTCCTGCAAGCCAATTTTGTC-3’

R 5’-TCTTCTCACCCTTCTTTTTCATTGT-5’

*IFNB* F 5’-TTGTGCTTCTCCACTACAGC-3’

R 3’-CTGTAAGTCTGTTAATGAAG-3’

*IFNA2* F 5’-CCTGATGAAGGAGGACTCCATT-3'

R 5’-AAAAAGGTGAGCTGGCATACG-3’

*IRF7* F 5’-GAGCCCTTACCTCCCCTGTTAT-3’

R 5’-CCACTGCAGCCCCTCATAG-3’

*IL23A* F 5’-TTCTGCTTGCAAAGGATCCA-3’

R 5’-TCCGATCCTAGCAGCTTCTCA-3’

*CXCL9*  F 5’-GTGCAAGGAACCCCAGTAGTGA-3’

R 5’-TAGTCCCTTGGTTGGTGCTGAT-3’

*GAPDH*  F 5’-ACAGTCCATGCCATCACTGCC-3’

R 5’-GCCTGCTTCACCACCTTCTTG-3’

*HTLVpx*  F 5’-CAAAGTTAACCATGCTTATTATCAGC-3’

R 5’-ACACGTAGACTGGGTATCCGAA-3’

| **List of reference numbers corresponding to the human primers used (Taq Man)** |
| --- |

*CD48* Hs00381156

*LCP2* Hs00175501

*CDC7* Hs00177487

*IFNG* Hs00174143

*SOCS1* Hs00705164

*IKBKAP* Hs00175353

*FYTTD1* Hs00260265

*IKBKG*  Hs00415849

*IDE*  Hs00610438

*AIM2* Hs00175457

*TNFSF7*  Hs00174297

*TNFSF11* Hs00243522

*ENG*  Hs00164438

*IL7*  Hs00174202

*GAPDH*  Hs99999905
